# Relationship Between Occlusal Factors and Temporomandibular Disorders: A Systematic Literature Review

**DOI:** 10.7759/cureus.54130

**Published:** 2024-02-13

**Authors:** Roberta Lekaviciute, Albertas Kriauciunas

**Affiliations:** 1 Faculty of Odontology, Medical Academy, Lithuanian University of Health Sciences, Kaunas, LTU; 2 Faculty of Odontology, Clinic of Dental and Maxillofacial Orthopaedics, Medical Academy, Lithuanian University of Health Sciences, Kaunas, LTU

**Keywords:** temporomandibular joint (tmj) disorders, temporomandibular disorders, malocclusion, tooth loss, bruxism, tmd, tmj

## Abstract

Temporomandibular disorders (TMD) originate from various components within the temporomandibular joint (TMJ), causing an impact on the masticatory muscles, the joint itself, and associated structures. They are a widely prevalent issue across the world. According to epidemiological research, up to 50% of adults in the population have TMD-related symptoms. The objective of this work was to analyze the existing scientific literature regarding the association between malocclusion classes, bruxism, and tooth loss in relation to the etiology of TMD. This systematic review was conducted following the Preferred Reporting Items for Systematic Reviews and Meta-Analyses (PRISMA) 2020 analysis protocol. For the development of the question focus, the population, intervention, control, and outcomes (PICO) study design protocol was used. The question in focus according to the PICO format was: “Do malocclusion, tooth loss, and bruxism contribute to temporomandibular disorders?”. The review was performed with articles from PubMed, Web of Science, and Google Scholar databases according to the specified inclusion and exclusion criteria. The included articles were not older than five years. The risk of bias was assessed in the included studies by using the Cochrane Risk-of-bias 2 (RoB-2) tool. Out of a total of 32 results received, 21 articles were chosen according to the established criteria after conducting a review and analysis of their full texts. The article search sequence was presented in the PRISMA 2020 flow diagram, and the outcomes of the chosen articles were presented. The literature results revealed a relationship between occlusion and the development of TMD. The influence of occlusal factors on the TMJ was explained by an examination of joint anatomy and symptoms related to TMD. This study revealed variations in TMJ factors across different malocclusion classes. Additionally, it was observed that the occurrence and attributes of TMD are influenced by the number of tooth loss quadrants and the frequency of missing teeth. Furthermore, a correlation was found between bruxism and the symptoms of TMD, including myofascial pain, disc displacement, arthralgia, and muscle disorders. This literature review provides comprehensive information on the relationship between malocclusion classes, bruxism, tooth loss, and TMDs. This prompts healthcare professionals to prioritize patients’ occlusal assessment and TMJ condition.

## Introduction and background

The temporomandibular joint (TMJ) is recognized as one of the most important joints in the human body due to its significant functions in dental occlusion and the neuromuscular system [1]. The TMJ complex is composed of various structures such as bone, cartilage, muscles, ligaments, and neurovascular channels that provide nourishment to these components [2]. Temporomandibular disorders (TMDs) arise from various structures within the TMJ. It is a condition that affects the masticatory muscles and the whole TMJ region structures, such as the mandible, muscle tissues, tendons, dental arches, and salivary glands, as well as the hyoid bone and the muscles that connect the latter to the scapula and the sternum, the muscles of the neck. Temporomandibular joint dysfunctions are characterized by joint and muscular pain, joint sounds, and restricted or irregular mandibular function [3].

This condition is a substantial public health issue, as it is the primary cause of non-dental orofacial pain and the second most frequent musculoskeletal ailment, following chronic low back pain [4]. Several epidemiological studies have demonstrated that adolescents frequently exhibit symptoms of TMD, along with parafunctional oral habits that are commonly observed in this age group [5]. Typically, TMDs are thought to impact around 5% to 15% of the adult population, but it has been reported that TMD-related symptoms are found in as many as 50% of adults [6]. However, only around 2% of those who have it need any form of intervention or treatment [7]. A considerable number of patients experience TMDs and either cope with them on their own or manage their symptoms independently [8].

The etiology of TMD is believed to be intricate and multifactorial, with many different factors potentially playing a role in its development [3-8]. Temporomandibular disorders are caused by a combination of various factors, including biomechanical, neuromuscular, biopsychosocial, and neurobiological factors [3], such as dental loss, dental wear, maladaptive dentures, cavities, improper restorations, premature contact of restorations, the inclination of teeth toward the space created by the tooth loss, bruxism, nail-biting, hand-jaw support, digit or pacifier sucking, as well as traumatic or degenerative lesions of the TMJ [9]. These factors are divided into three categories: predisposing factors, including structural, metabolic, and psychological conditions; initiating factors, including trauma or repetitive adverse loading of the masticatory system; and aggravating factors, including parafunctional, hormonal, or psychosocial factors [10]. 

There has been debate around the role of occlusion in TMD, and this topic has been the subject of various studies and discussions. Some authors argue that occlusion is the main factor in TMD symptoms, while others claim that it has no involvement and that behavioral, psychological, and neurological factors are the fundamental causes of TMD [11]. During the 1960s and 1970s, functional disorders of the masticatory system were primarily attributed to occlusion and emotional stress. However, in the 1970s, there was a significant increase in attention to pain disorders originating from intracapsular sources, which were discussed and described by Farrar and McCarty [1]. In 2017, Manfredini et al. conducted a systematic review where they described “the end of an era” and advised clinicians to abandon the old gnathological paradigm in TMD practice. They encouraged clinicians to adopt new approaches to evaluate and treat TMD [12].

In this study, we aimed to access the most recent scientific findings concerning the relationship between TMDs and malocclusions, tooth loss, and bruxism.

## Review

Materials and methods

Aim of the Literature Review

This literature review aimed to examine the current scientific literature findings associating TMDs’ relationship with malocclusion, tooth loss, and bruxism.

Inclusion and Exclusion Criteria

The inclusion and exclusion criteria for the selection of articles are described in Tables 1-2.

**Table 1:** Inclusion criteria

Article inclusion criteria:
• All published study designs except case reports and literature reviews
• Articles in which patients were affected by malocclusion, bruxism, or tooth loss
• Research on patients who were affected by malocclusion, bruxism, or tooth loss and had their temporomandibular disorder symptoms analyzed
• Articles on malocclusion, bruxism, or tooth loss and their association with temporomandibular disorders
• Laboratory studies and clinical studies regarding the impact of occlusal factors on temporomandibular disorders
• Articles that are written in English and were published within the last five years

**Table 2:** Exclusion criteria

Article exclusion criteria:
• Articles that do not cover malocclusion, bruxism, or the association of tooth loss with temporomandibular disorders
• Research that does not include patients with malocclusion, bruxism, tooth loss, or temporomandibular disorders
• Articles examining temporomandibular disorders without analyzing the occlusal factors of the patients
• Articles that include patients with malocclusion, bruxism, or tooth loss but do not analyze temporomandibular disorders
• Pilot studies, case reports, systemic or literature reviews, and meta-analyses
• Articles that are written in languages other than English and are older than five years

Information Sources and Search Strategy

A systematic literature search was conducted according to the Preferred Reporting Items for Systematic Reviews and Meta-Analyses (PRISMA) guidelines for clinical trials and literature analysis published between 2019 and 2023 [13]. The electronic literature searches were performed in the databases of PubMed, Web of Science, and Google Scholar. Databases were searched using different combinations of the following keywords: “temporomandibular disorder," "TMD," “occlusal factors," "bruxism," “tooth loss," “missing teeth," “pathological occlusal changes," and “TMJ pathology”.

For the development of the question focus, the population, intervention, control, and outcomes (PICO) study design protocol was used. The question in focus according to the PICO format was: “Do malocclusion, tooth loss, and bruxism contribute to temporomandibular disorders?”.

The selected articles passed four stages: 1. selection by the relevant article title; 2. duplicate removal; 3. selection by the relevance of the abstract; and 4. full-text analysis.

The titles and abstracts were analyzed by the two authors (Lekavičiūtė R and Kriaučiūnas A), followed by the selection of complete articles for careful reviewing and analysis according to the eligibility criteria. Reviewers collected data from each report. The assessment of potential bias was conducted using the guidelines outlined in the Cochrane Risk-of-bias 2 (RoB-2) tool [14]. 

Study Selection

**Figure 1:**
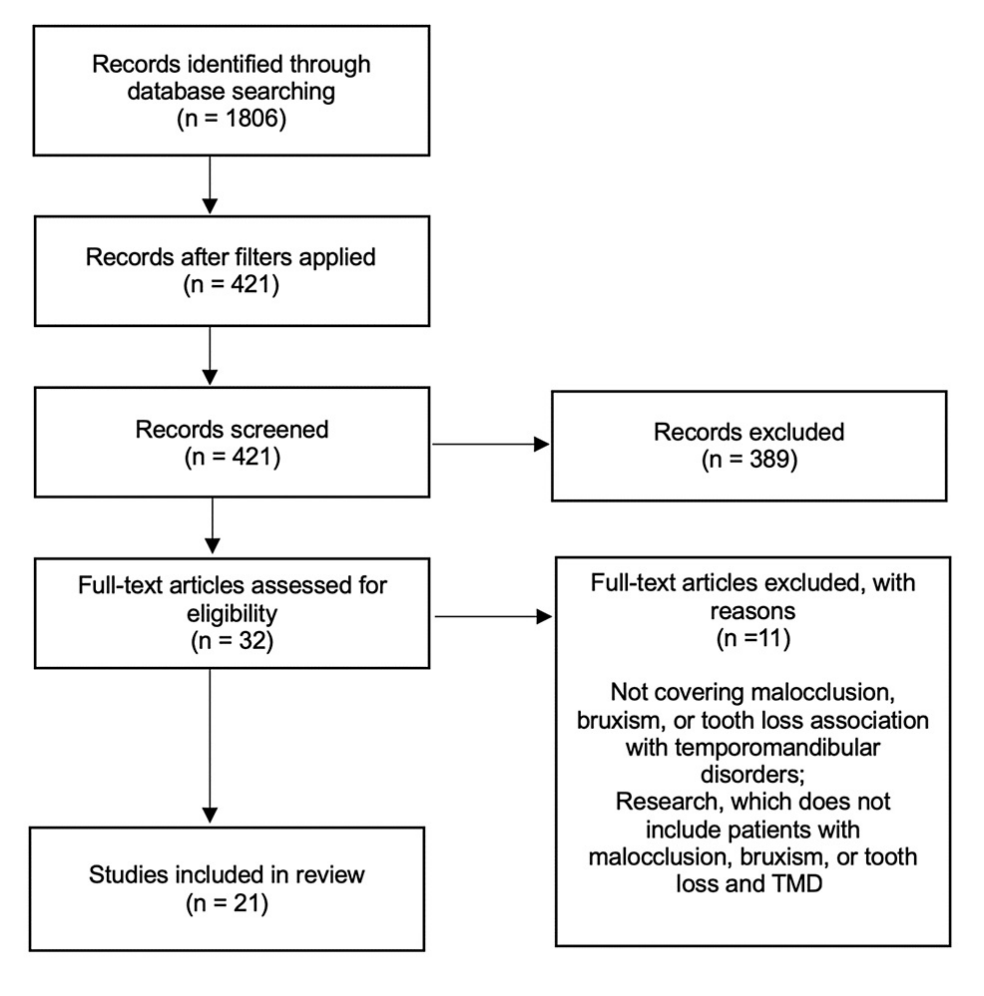
A PRISMA flowchart outlining the study selection process PRISMA: Preferred Reporting Items for Systematic Reviews and Meta-Analyses; TMD: temporomandibular disorder

The findings were synthesized using the guidelines outlined in the PRISMA 2020 statement, an updated framework for reporting systematic reviews [13]. The identification of records was initially conducted by performing a search in a database, resulting in a total of 1,806 records. After the inclusion criteria for articles were applied, 421 records remained suitable for screening. A total of 389 records were eliminated from the screening process since they were deemed irrelevant to the topic. A total of 32 full-text papers were evaluated to determine their eligibility for inclusion in the study. Of these, 11 articles were removed based on the following criteria: lack of coverage of the link between malocclusion, bruxism, or tooth loss and TMD; and studies that did not involve patients with malocclusion, bruxism, or tooth loss and TMD. A total of 21 studies were included in the review. Figure 1 provides a visual depiction of the search technique and the synthesis of results.

Characteristics of the Studies

Seven studies assessed the relationship between different dental malocclusions and TMJ morphology [15-21]. In six studies, the three-dimensional position and morphology of the condyle in the glenoid fossa were assessed using cone-beam computed tomography (CBCT) [15-17, 19-21]. Other studies used lateral X-rays [20] and magnetic resonance imaging (MRI) [18]. The studies involved a total of 608 subjects. Six of the studies included in the research analyzed a group of patients with malocclusion, including those with class III [15, 16, 19], class II division 1 [18, 19, 21], and class II division 2 [18, 19, 21], and compared them to non-malocclusion individuals with class I [15, 16, 18, 19, 20, 21]. Additionally, one study compared two different types of malocclusions, specifically class II division 1 and division 2 [17]. The main focus of all these studies was to compare the morphological characteristics of the TMJ between individuals with different types of malocclusion and healthy subjects without malocclusion.

Eight studies were conducted to investigate the association between dental loss and TMDs, with a total of 2008 subjects being included [22-29]. All studies, except one [27], employed a retrospective study design that compared patients with missing teeth to those without missing teeth. Among these studies, four utilized orthopantomograms [22, 23, 28] and CBCT [24] to analyze the articular eminence inclination (AEI). In two studies, questionnaires were used to investigate the effect of tooth loss on the TMJ [25, 27], while in three studies, clinical examination was used to evaluate TMD symptoms [26, 28, 29].

There were a total of 1,257 participants in six studies [30-35] that examined the effects of bruxism on TMD. Two of the studies applied the Research Diagnostic Criteria for Temporomandibular Disorders (RDC/TMD) [30, 31], whereas three of the studies employed the Diagnostic Criteria for Temporomandibular Disorders (DC/TMD) for the diagnosis of TMD [32, 33, 35]. Several studies examined the effect of bruxism on the TMJ using questionnaires [30, 35], self-reports [31, 34], and clinical examination results [30, 31, 33, 34].

Types of Studies

The literature review includes a diverse set of studies, including cross-sectional, retrospective, descriptive-analytic, single-center, case-control, interdisciplinary longitudinal, and clinical studies. We found cross-sectional studies [16-18, 21, 22, 28, 29, 33], retrospective cross-sectional studies [15, 19, 20, 23, 24], descriptive-analytic with a cross-sectional design [25], case-control studies [26, 35], a single-center retrospective clinical study [27], an interdisciplinary longitudinal study [30], a clinical study [31], and retrospective clinical studies [32, 34].

Risk of Bias Assessment Within Studies

**Figure 2:**
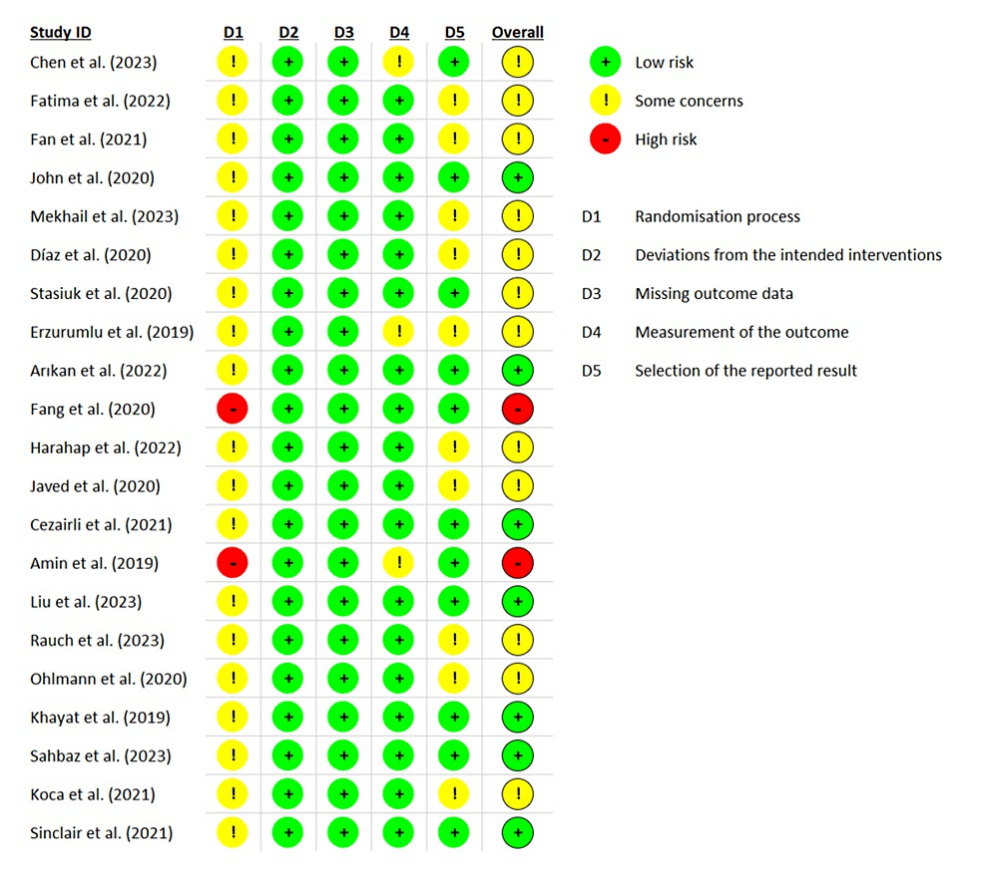
Overall results of the Cochrane Risk-of-Bias analysis, with bias in each domain expressed for all included studies in this review.

**Figure 3:**
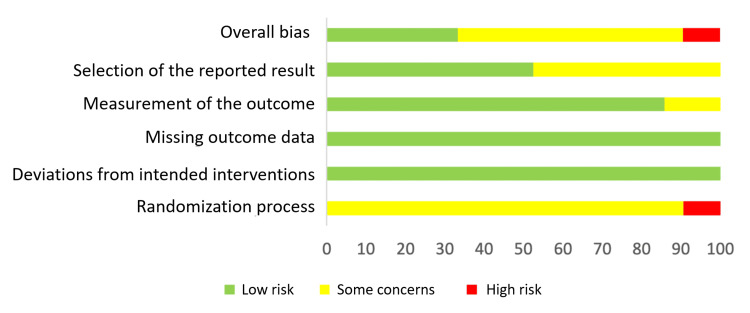
Results of the analysis of bias conducted using the Cochrane Risk-of-bias 2 (RoB-2) tool.

The assessment of potential bias was conducted using the guidelines outlined in the Cochrane Risk-of-bias 2 (RoB-2) tool [14]. The analysis of the evaluated research indicated that the overall reported bias was low for 33.3% of the total studies examined. Furthermore, it was seen that a certain percentage (57.2%) of the studies evaluated some concerns, while a proportion (9.5%) of these studies were determined to have a high bias risk (Figures 2-3).

Results

Summary of Studies Based on Malocclusion

Chen et al. studied 90 patients who had class III malocclusion and compared the results with 30 class I normodivergent patients [15]. The participants in the study group were subdivided into three groups based on their vertical skeletal pattern: hypodivergent, normodivergent, and hyperdivergent, comprising 30 patients in each category [15]. Measurements were conducted on CBCT images, and a total of 240 TMJs were measured and examined. Based on the results of the study, it was observed that patients belonging to the class III normodivergent and hyperdivergent groups displayed a relatively less deep glenoid fossa (P=0.004; P=0.001), a wider articular fossa, thicker mandibular eminence (P=0.046), flatter AEI (P=0.012; P=0.001) and anterior inclination of the mandibular condyle (AIC) (P=0.003; P=0.001) [15]. Furthermore, it was noted that individuals affected by skeletal class III malocclusion exhibited a noticeably inferiorly positioned condyle [15]. The study findings did not reveal any significant difference in the morphology of the TMJ between the left and right sides [15].

The same comparison between class III malocclusion and class I was made in research conducted by Fatima et al. The study analyzed the three-dimensional position and morphology of the condyle in the glenoid fossa on CBCT images [16]. The researchers conducted a detailed examination of the TMJ structures and measured various parameters to evaluate the differences between the two occlusal groups. The study findings showed that patients with class III malocclusion exhibited a statistically significant reduction in the anterior (p=0.048) and superior (p=0.045) joint spaces when compared to those with class I occlusion, while other parameters were statistically similar between the groups [16].

A study conducted by Fan et al. analyzed 67 individuals with three different malocclusions: class I (n=24), class II division 1 (n=20), and class II division 2 (n=23) [17]. The CBCT was used to evaluate a total of 134 TMJs, and it showed that AEI varied significantly between the two classes, with class II division 2 displaying the highest AEI (p=0.017) [17]. Furthermore, individuals with class II division 2 malocclusion exhibited a greater tendency for inward rotation of the condyles. Additionally, there was no significant difference in the shape of the glenoid fossa between the left and right sides (p >0.05) [17].

John et al. conducted a study that assessed the same divisions of class II malocclusion as the previous research, but this time using MRI for their assessments [18]. Based on the results of the study, it was found that class II division 2 patients had significantly higher mean values for the articular disk position in the sagittal and coronal planes, condylar concentricity, and anterior and posterior joint spaces (p < 0.001) [18]. On the other hand, Mekhail et al.’s research found that there was no observed difference in TMJ morphology between class II division 1 and division 2 in a different study (p >0.05) [19].

Díaz et al. included 92 patients with class II malocclusion and compared the results with those of 96 patients with class I malocclusion. The study used CBCTs to evaluate the height and width of the condyle, the mediolateral and anteroposterior dimensions of the condyle, as well as the shape of the glenoid fossa, while lateral radiographs were used to evaluate other measurements, such as the A point, nasion, B point (ANB) angle and the Wits appraisal [20]. The results of the study revealed that individuals with class II malocclusion exhibited significantly smaller mediolateral (p <0.001) and anteroposterior (p=0.021) dimensions of the condyle, as well as decreased condylar height (p=0.005) and neck width (p=0.003) in comparison to those with class I malocclusion [20].

A further research project performed by Stasiuk et al. included 143 individuals with different malocclusions: class I (n=34), class II (n=30), and class III (n=6) [21]. Depending on the period of bite development, 30 patients had mixed occlusion and 40 had permanent occlusion. All participants underwent CT examinations to ascertain the position of the TMJ heads in the midsagittal plane of the joint using the H. Gelb method [21]. The analysis of the symmetrical position of the TMJ heads in the 4/7 position across various malocclusion classes revealed that only 11.43% of cases exhibited such symmetry, regardless of the occlusal pathology class, highlighting a strong association between dentofacial abnormalities and TMJ pathology [21]. Synthesis and analysis of findings (PICO) on the investigation of the correlation between malocclusions and TMD are represented in Table 3.

**Table 3:** Reported studies on the investigation of the correlation between malocclusions and temporomandibular disorders

Study	Population	Intervention	Comparison	Outcomes
Chen et al. [15], 2023	N=120; two groups: class III (n=90) and class I (n=30)	Cone-beam computed tomography (CBCT)	The position and morphology of the temporomandibular joint (TMJ)	Class III normodivergent and hyperdivergent groups had a shallower glenoid fossa, a wider articular fossa, and a larger mandibular eminence inclination. In class III malocclusion, the condyle was positioned lower. No TMJ component differences were found between the left and right sides.
Fatima et al. [16], 2022	N=40; four groups (n=10): class I, class II/1, class II/2, and class III	CBCT	The position and morphology of the condyle in the glenoid fossa	Class III malocclusion showed significantly smaller anterior and superior joint space compared to class I.
Fan et al. [17], 2021	N=67; three groups: class I (n=24), class II/1 (n=20), and class II/2 (n=23)	CBCT	Angular and linear measurements of TMJ	The articular eminence inclination (AEI) differed significantly between class I and class II, with class II/2 showing the highest AEI. Class II/2 has a greater tendency for inward condyle rotation. The glenoid fossa shape is similar on both sides, but it is not reliable for identifying malocclusions.
John et al. [18], 2020	N=75; three groups: class I (n=25), class II vertical (n=25), and class II horizontal (n=25)	MRI	Articular disk position, condylar position, and joint spaces	The class II vertical group had the highest mean articular disk position in both the sagittal and coronal planes and the highest mean condylar concentricity. The class II vertical group had significantly higher mean values for both the anterior and posterior joint spaces.
Mekhail et al. [19], 2023	N=48; two groups: class II/1 and class II/2	CBCT	TMJ morphological characteristics	TMJ morphology showed no significant differences between class II/1 and class II/2.
Díaz et al. [20], 2020	N=188; two groups: class II (n=92) and class I (n=96)	CBCT and lateral radiographs	TMJ morphological characteristics	Class II had smaller condyle dimensions, as well as smaller condyle height and neck width. The A point, nasion, B point (ANB) angle was the main factor influencing a decrease in condyle dimensions in class II.
Stasiuk et al. [21], 2020	N=70; three groups: class I (n=34), class II (n=30), and class III (n=6)	CT	The position of the temporomandibular joint heads	The symmetrical position of TMJ heads in the 4/7 position was observed in only 11.43% of cases. The percentage of patients in the 4/7 position decreased from 17.65% in class I to 6.67% in class II, with no patients observed in class III.

Summary of Studies Based on Tooth Loss

In one of the studies, Erzurumlu et al. assessed panoramic images of 50 edentulous patients and 50 dentate patients to measure the angle of the AEI in both TMJs [22]. The findings revealed a significant correlation between dental loss and changes in the angle of the AEI, where the AEI was higher in dentate patients (42.6 ± 4.3º) compared to edentulous patients (35.1 ± 4.7º) (p <0.001) [22]. The impact of the duration of edentulousness on the degree of AEI values was also analyzed. However, no positive correlations were observed between the duration of edentulousness and the values of AEI (p=0.782) [22]. 

In a study conducted by Arıkan et al., the effect of edentulousness on eminence inclination, joint space, and condyle head widths was investigated. The study consisted of 24 completely toothless patients, whose results were compared with those of 24 dentulous patients [23]. The measurements were made using CBCT images. The results of the study showed that there was a statistically significant difference in the mean AEI between dentulous (47.51º) and edentulous patients (41.87º) (p=0.001). In addition, there was a significant decrease in the mediolateral (p=0.020) and anteroposterior (p=0.000) dimensions of the condyle head, while no significant difference was observed in the anterior (p=0.299), superior (p=0.067), or posterior joint space (p=0.803) in both dentulous and edentulous patients [23].

Fang et al. split 109 patients into two groups: patients without missing teeth (n=20) and patients with unilateral one or more than one posterior tooth loss (n=89) [24]. The panoramic analysis revealed that the patients without missing teeth (46.66º) had a higher AEI compared to the patients with missing teeth (42.28º) (p <0.001) [24]. On the other hand, Harahap and Chairunnisa’s research compared TMJ morphological characteristics between patients who lost three or more teeth and those who lost less than three teeth. The number of tooth losses (p=0.023), as well as the number of tooth loss quadrants, exhibited a significant correlation with TMD (p=0.016) [25]. This study failed to find any significant association between the number of lost teeth and AEI (p >0.05) [25]. However, a separate analysis demonstrated a significant correlation between the number of tooth loss quadrants and the inclination of the articular eminence on the right side (p=0.017), while no such correlation was observed on the left side (p=0.577) [25]. 

Further research conducted by Javed et al. included 185 patients who were asked to fill out a questionnaire consisting of 10 questions related to common symptoms of TMD to evaluate the effect of dental loss on the TMJ [26]. A case-control study was conducted with comparison groups of TMD patients versus TMD-free patients, revealing a significant correlation between the number of tooth loss quadrants and TMD, indicating that an increase in the number of quadrants with tooth loss can increase the risk of TMD (p <0.001). However, the number of teeth lost itself did not show any association with TMD (p=0.056) [26].

A study performed by Cezairli et al. analyzed 212 patients who had various TMJ-related symptoms, such as pain, limited mouth opening, joint sounds, and difficulty chewing [27]. Three different groups were formed: individuals without missing teeth (n=96), one to five missing teeth (n=81), and more than six missing teeth (n=35) [27]. The results of the clinical examination demonstrated a significant correlation between the number of missing teeth and symptoms of pain (p=0.001), disability during chewing (p <0.001), maximum mouth opening (MMO) (p=0.003), and TMJ sound (p=0.008). Nevertheless, there was no significant correlation between the number of quadrants with missing teeth and locking (p=0.392) [27].

Another research project conducted by Amin et al. studied the TMD symptoms of 143 individuals with more than two missing teeth. Detailed intraoral and extraoral examination results revealed a significant difference between the number of missing teeth and the frequency of TMD symptoms, including muscle tenderness, restricted mouth opening, and TMJ sound, excluding mandibular deviation (p=0.068) [28]. The frequency of TMD signs was observed to increase with the number of missing teeth, with patients with more than 20 missing teeth demonstrating the highest frequency. Furthermore, TMJ sound was identified as the most common TMD sign among this patient population (39.90%) [28].

Liu et al. conducted a study to examine the association between congenitally missing teeth and TMD. The analysis included 583 patients with one or more congenitally missing non-third molar teeth and 586 controls with no congenitally missing non-third molar teeth [29]. To diagnose TMD, the researchers utilized the DC/TMD guidelines. Participants with congenitally absent teeth had a considerably higher prevalence of TMD (67.24%) than those without congenitally absent teeth (45.90%) (p <0.001) [29]. In addition, congenitally absent teeth were significantly associated with TMD overall, intra-articular TMD, and pain-related TMD (p <0.001) [29]. Synthesis and analysis of findings (PICO) on the investigation of the correlation between tooth loss and TMD are represented in Table 4. 

**Table 4:** Reported studies on the investigation of the correlation between tooth loss and temporomandibular disorders

Study	Population	Intervention	Comparison	Outcomes
Erzurumlu et al. [22], 2019	N=100; two groups: dentate (n=50) and edentulous patients (n=50)	Orthopantomogram (OPG)	Articular eminence inclination (AEI)	The average AEI value was higher in the dentate group. There were no AEI differences between males and females in the dentate group, but males had higher AEI values in the edentulous group. No correlations were observed between the duration of edentulousness and the AEI values.
Arıkan et al. [23], 2022	N=48; two groups: dentulous (n=24) and edentulous patients (n=24)	Cone-beam computed tomography (CBCT)	Articular eminence inclination, condyle head widths, and joint space	The AEI value, mediolateral and anteroposterior width of the condyle were significantly higher in the dentulous group. No notable differences were observed between both groups in terms of the anterior, superior, and posterior joint space.
Fang et al. [24], 2020	N=109; two groups: non-missing teeth (n=20) and missing teeth groups (n=89)	OPG	Articular eminence inclination, craniocervical angle	The non-missing teeth group exhibited a higher angle of AEI. The missing teeth group displayed a smaller craniocervical angle. The missing teeth group had a lower number of occlusal planes passing through the intersection of the first and second cervical vertebrae.
Harahap et al. [25], 2022	N=42; two groups: ≥3 missing teeth (n=19) and <3 missing teeth (n=23). Four groups (number of quadrants): 1 (n=10), 2 (n=8), 3 (n=8), and 4 (n=16)	OPG, Fonseca’s questionnaire	Articular eminence inclination	The number of tooth losses and tooth loss quadrants showed a significant relationship with temporomandibular disorder (TMD). The relationship between the number of tooth losses and AEI was not significant, except for the right-side AEI, which showed a significant association with the number of tooth loss quadrants.
Javed et al. [26], 2020	N=185; two groups: TMD patients (n=95) and TMD – free patients (n=190). 2 groups: ≥5 missing teeth (n=104) and <5 missing teeth (n=181)	The Conti Anamnestic Questionnaire	TMD symptoms and the number of lost teeth	A significant correlation exists between the number of tooth loss quadrants and TMD. More tooth loss quadrants are associated with an increased risk of TMD. The number of teeth lost alone does not show any association with TMD.
Cezairli et al. [27], 2021	N=212; three groups: No missing teeth (n=96), 1–5 missing teeth (n=81), and ≥6 missing teeth (n=35)	The visual analog scale, the electronic caliper, a stethoscope, and clinical examination.	TMD symptoms: maximum mouth opening (MMO), joint sounds, pain, disability in chewing and locking	Age, pain, temporomandibular joint (TMJ) sound, and chewing disability showed significant variations based on the number of missing teeth. Gender, MMO, and locking did not exhibit significant differences in relation to the number of missing teeth.
Amin et al. [28], 2019	N=143; one group: >2 missing teeth (n=143)	Intraoral and extraoral examination	TMD symptoms: tenderness of muscles, restricted mouth opening, TMJ sound, and mandibular deviation	The most common symptom of TMD was the TMJ sound. The increasing number of missing teeth was associated with a higher frequency of TMD signs. Patients with more than 20 missing teeth had the highest rate of TMD signs. All TMD signs, except mandibular deviation, showed statistically significant associations with the number of missing teeth.
Liu et al. [29], 2023	N=1,169; two groups: Congenitally missing teeth (n=583) and non-congenitally missing teeth (n=586)	The clinical examination adhered to the Diagnostic Criteria for Temporomandibular Disorders (DC/TMD) Axis I and Wieckiewicz’s protocol.	TMD symptoms: disc displacement, degenerative joint disease and dislocation, myalgia, myofascial pain with referral, arthralgia, and headaches	Participants with congenitally missing teeth exhibited a significantly higher prevalence of overall TMD. Congenitally missing teeth showed a significant association with overall TMD, intra-articular TMD, and pain-related TMD.

Summary of Studies Based on Bruxism

A study conducted by Rauch et al. analyzed 191 senior individuals. The participants completed a questionnaire and underwent a clinical examination based on the RDC/TMD guidelines [30]. During the clinical examination, it was observed that 27.2% of the participants displayed indications of tooth wear [30]. The findings indicated that bruxism was present in a quarter of the elderly population, with 15.2% receiving a single diagnosis and 1.6% receiving multiple diagnoses, which included disc displacements (9.4%) and degenerative joint diseases (8.9%) [30].

Another study conducted by Ohlmann et al. included 110 participants. Different groups were formed: non-bruxers (n=52) and bruxers (n=58), with the latter group further categorized into moderate bruxers (n=23) and severe bruxers (n=35) [31]. The presence of sleep bruxism (SB) was determined based on self-reporting, a clinical examination using the Axis-I protocol of the RDC/TMD to evaluate bruxism signs, and data from a portable electromyography (EMG)/electrocardiogram (ECG) recorder [31]. The findings suggest that bruxers are more likely to be diagnosed with myofascial pain (RDC/TMD group-I) (p=0.011), but there were no significant differences between bruxers and non-bruxers in terms of diagnoses of disc displacement (RDC/TMD group-II) (p=0.930) and arthralgia, arthritis, or arthrosis (RDC/TMD group-III) (p=0.789) [31].

Khayat et al. split 494 patients into 4 groups: 177 patients with painful TMD, 64 patients with disc displacement, 64 individuals with both painful TMD and disc displacement, and 149 patients without TMD [32]. For the determination of TMD, the DC/TMD was used, and for the diagnosis of SB, a multifactorial approach was implemented, including the Oral Behavior Checklist, participant self-reporting, and evaluation of dental wear levels [32]. The study revealed a statistically significant correlation between painful TMD and bruxism (SB and awake bruxism (AB)) (p <0.05), although no significant association was found between disc displacement and both SB and AB (p=0.111 and p=1.687, respectively) [32]. 

A further research project conducted by Sahbaz et al. included 143 patients. Following a physical examination, the participants were categorized into three groups based on their diagnosis of bruxism: SB (n=25), AB (n=42), and non-bruxers (n=76) [33]. All study participants underwent an evaluation using DC/TMD [33]. The study findings showed that individuals with AB exhibited a significantly higher prevalence of arthralgia than non-bruxers (p=0.021), while both SB and AB were associated with significantly higher frequencies of muscle disorders and disk displacement with reduction when compared to non-bruxers (p <0.05) [33]. Assessment of jaw functional limitation was measured using the Jaw Functional Limitational Scale (JFLS-8) scoring system, while the TMD Pain Screener was used to identify individuals experiencing pain related to TMD. The results showed that scores were significantly higher in patients with AB than those with SB and non-bruxers (p <0.05) [33].

Koca et al. included 167 patients affected by bruxism and compared the results with those of 112 patients without bruxism. The diagnosis of probable bruxism was determined via self-reports and clinical examination results. Magnetic resonance imaging was used to evaluate TMJ morphological characteristics [34]. The results of the TMJ MRI findings did not show any significant difference between the control group and the bruxism group (p >0.05) [34]. However, significant dissimilarities were found in disc position (p=0.822), disc form (p=0.156), condyle shape (p=0.953), and effusion (p=0.975) between the sides affected by pain and non-painful sides in patients without bruxism and bruxers [34].

Sinclair et al.’s investigation revealed that in patients affected by SB, 46.4% exhibited a co-occurring diagnosis of TMD [35]. However, the results from the chi-square test did not demonstrate a statistically significant correlation between TMD and sleep bruxism (p=0.675). Additionally, analysis of the participants using the DC/TMD classification system revealed that local myalgia was the most common subtype of TMD observed in the sample of 28 individuals (71.4%) [35]. The next most common subtypes were arthralgia with disc displacement without reduction without limitation of opening (7.1%) and disc displacement with reduction (DDwR) without opening limitation (7.1%). Local myalgia plus DDwR without limitation of the opening was observed in 7.16% of individuals. Headaches attributed to TMD and DDwR without limitation of opening associated with headaches attributed to TMD were observed in 3.6% of the participants [35]. Synthesis and analysis of findings (PICO) on the investigation of the correlation between bruxism and TMD are represented in Table 5.

**Table 5:** Reported studies on the investigation of the correlation between bruxism and temporomandibular disorders

Study	Population	Intervention	Comparison	Outcomes
Rauch et al. [30], 2023	N=191; two groups: born between 1950 and 1952 (n=121), and born between 1930 and 1932 (n=70)	A questionnaire, and clinical examination according to the Research Diagnostic Criteria for Temporomandibular Disorders (RDC/TMD) guidelines	Bruxism symptoms: indentations on the tongue, linea alba, and visual assessment of tooth wear	15.2% of patients received a single diagnosis, while 1.6% received multiple diagnoses (disc displacement (DD), degenerative joint diseases). A total of 24.7% reported experiencing bruxism (11.9% awake bruxism (AB) and 16.2% sleep bruxism (SB)); 27.2% of patients had clinical tooth wear. The prevalence of probable bruxism was significantly higher in patients born between 1930 and 1932.
Ohlmann et al. [31], 2020	N=110 2 groups: bruxers (n=58) and non-bruxers (n=52); two groups: moderate (n=23) and severe bruxers (=35)	Self-reporting of bruxism, a clinical examination based on the Axis-I protocol of the RDC/TMD, data from a portable electromyography (EMG)/electrocardiogram (ECG) recorder recorder.	Temporomandibular disorder (TMD) symptoms: muscle disorders, DD, arthralgia, osteoarthritis, osteoarthritis; Bruxism symptoms: tooth wear, impressions of teeth, and hypertrophy of the masseter muscle	Ten bruxism patients received a diagnosis of myofascial pain, while none of the non-bruxers received this diagnosis. No significant differences were observed between bruxers and non-bruxers in terms of RDC/TMD group-II (disc displacement) and group-III (arthralgia, arthritis, and arthrosis) diagnoses. Bruxers exhibited a significantly higher prevalence of somatization.
Khayat et al. [32], 2019	N=494; four groups divided as follows: TMD-free (n=149), Pain-TMD (n=177), DD (n=64), and Pain-TMD+DD group (n=104)	Diagnostic Criteria for Temporomandibular Disorders (DC/TMD) guidelines, clinical examination according to the Oral Behavior Checklist (OBC), and dental wear level.	TMD symptoms: myalgia, myofascial pain, headache, arthralgia, and DD	SB showed a significant association with pain-related TMD, but not with DD. SB had a greater impact on Pain-TMD, and the interaction between Pain-TMD and DD was more influenced by SB than by Pain-TMD alone. AB was significantly associated with both Pain-TMD and Pain-TMD + DD, but not with DD alone.
Sahbaz et al. [33], 2023	N=143; three groups divided as follows: sleep bruxism (SB) (n=25), awake bruxism (AB) (n=42), and non-bruxers (n=76); two groups divided as follows: TMD (n=79) and TMD-free patients (n=64)	DC/TMD guidelines (TMD pain screener, symptom questionnaire, and clinical examination)	Pain-related TMD, headaches, and intra-articular joint disorders	Muscle disorders and DD were more frequent in individuals with bruxism. The AB group had a higher prevalence of arthralgia compared to non-bruxers. The AB group showed higher Jaw Functional Limitation Scale and TMD Pain Screener scores than other groups. Bruxers had higher distress levels, graded chronic pain, and OBC scores. Only the OBC score was significantly higher in the TMD subgroup.
Koca et al. [34], 2021	N=279; two groups: bruxers (n=167) and non-bruxers (n=112)	Self-reports and clinical examination, MRI	Temporomandibular joint (TMJ) morphological characteristics	There were no significant differences in disc/condyle relation, structure, or effusion. Disc position, form, condyle shape, and effusion differed significantly between painful and non-painful sides.
Sinclair et al. [35], 2021	N=40; 2 groups: SB (n=17) and non-bruxers (n=23); 2 groups: TMD (n=28) and TMD-free patients (n=12)	Polysomnography, European Academy of Craniomandibular Disorders (AEDC) questionnaire, the DC/TMD.	TMD symptoms: arthralgia, disc displacement, local myalgia, and headache	The rate of TMD in SB individuals was 46.4%. No significant association between TMD and SB was found. Among TMD patients, the most common TMD subtype was local myalgia.

Discussion

Multiple researchers have analyzed the association of different malocclusions, tooth loss, or bruxism with TMD signs and symptoms. Studies examining the link between occlusal factors and TMD have arrived at different conclusions. While some authors have found a strong association between TMD and occlusal factors, such as malocclusions, bruxism, or dental loss, others have reported a weak or no link between all these conditions. However, studies regarding TMDs are not limited to occlusal factors. The relationship between occlusion and TMD is still a controversial topic in dentistry.

In recent years, two consecutive reviews aimed to summarize all the findings on this topic, pointing out the challenges in drawing conclusions [4, 12]. In particular, a review conducted by Manfredini et al. in 2017 regarding the connection between dental occlusion and TMD revealed a lack of consensus among studies using diverse assessment methods for TMD. The findings of this literature review consistently indicate a limited clinically significant association between TMD and dental occlusion, suggesting that clinicians should consider moving away from the traditional gnathological paradigm in TMD treatment [12]. In a 2020 review by Michelotti et al., similar inconsistencies were found, suggesting that evidence from population-based surveys indicates a weak and inconsistent association between occlusion and TMD. This highlights the need for clinicians to reevaluate the role of occlusion in causing TMD, considering the interpretation of input at the central nervous system level and individual adaptability to prevent maladaptive behaviors. Additionally, dentists should possess knowledge about the multifactorial nature of TMD and proficiency in managing patients during dental procedures [4].

Several studies have found that occlusal factors do not play a significant role in the prevalence of TMD [36-38]. Research conducted by Aboalnaga et al. examined the dental and skeletal aspects of malocclusion in the anteroposterior and vertical dimensions among individuals with TMD. However, the results indicated that there were no significant associations between the occlusal variables and TMD parameters [36]. In another investigation by Manfredini et al., the presence of asymmetric molar or canine angle classes on both sides was explored for its potential association with TMD. The study concluded that such an association does not exist and that dental asymmetries have a minimal correlation with the presence of TMD signs and symptoms [37]. Research conducted by Ebadian et al. examined the relationship between occlusal factors, parafunctional habits, and TMD. The results revealed that, apart from bruxism, there were no correlations between other parafunctional habits and TMD [38]. Additionally, the investigated occlusal factors, including dental relationship, lateral occlusal scheme, horizontal differences between centric occlusion and maximum intercuspation (MI), and the difference between MI and mandibular resting position, did not show any significant correlations with TMD signs and symptoms [38].

Another factor found in the research that increases the prevalence of TMD is psychological. Cao et al. conducted a study that examined different subtypes of acute and chronic TMD and their relationship with psychological and sleep impairments [39]. The findings revealed that chronic pain-related TMDs were associated with elevated levels of psychological distress and poor sleep quality, whereas chronic intra-articular TMDs did not show the same association. Additionally, stress and depression were identified as factors that increased the probability of experiencing chronic pain-related TMD [39]. Research performed by Restrepo et al. focused on adolescents in rural and urban areas, and their findings indicated that pain-related TMDs were linked to psychological factors [40]. Symptoms of anxiety, depression, and somatization were found to be associated with TMD, even when the frequency of symptoms was not severe [40]. Another study conducted by Ye et al. emphasized the comorbidity of anxiety, depression, and high pain catastrophizing with TMD [41]. It was observed that all three psychological profiles were significant risk factors for TMD, with depression being the primary risk factor for pain-related TMD, while anxiety posed the highest risk for intra-articular TMD [41].

Several research studies have indicated that overall posture additionally plays a significant role in the prevalence of TMDs. A study by Hong et al. investigated the associations between degenerative changes in the cervical spine, head and neck postures, and myofascial pain in the craniocervical musculature among elderly individuals with myofascial TMD [42]. The study found that degenerative changes in the cervical spine were linked to altered head postures and the development of active myofascial trigger points in the craniocervical musculature in elderly individuals with myofascial TMD [42]. On the other hand, Ekici et al. conducted a study that examined the relationship between TMD and cervical posture, as well as the position of the hyoid bone [43]. The findings of this study revealed that TMDs are not associated with craniocervical posture but rather with the position of the hyoid bone and craniofacial morphology [43]. Another investigation by Fang et al. discovered that unilateral missing teeth are associated with changes in spine morphology and posture [24]. Their research findings indicated that unilateral tooth loss has the potential to decrease the cranio-cervical angle and increase the deviation of the occlusal plane from the C1-C2 intersection [24]. As a result, it leads to the disruptive posturing of C1 and C2, potentially affecting the balance of biomechanical and physiological development in the TMJ [24]. Nevertheless, a deeper analysis and further investigation into the impact of occlusal factors on TMDs are necessary in the future.

## Conclusions

After analyzing the articles, the following conclusions can be drawn: the factors related to the TMJ vary across different classes of malocclusion, thereby emphasizing the influence of malocclusion on temporomandibular joint disorders; the number of tooth loss quadrants and the frequency of missing teeth contribute to the prevalence and characteristics of TMDs; bruxism is associated with TMD symptoms such as myofascial pain, disc displacement, arthralgia, and muscle disorders.
